# Melatonin Protects Goat Spermatogonial Stem Cells against Oxidative Damage during Cryopreservation by Improving Antioxidant Capacity and Inhibiting Mitochondrial Apoptosis Pathway

**DOI:** 10.1155/2020/5954635

**Published:** 2020-12-31

**Authors:** Tian-Yu Feng, Qian Li, Fa Ren, Hua-Ming Xi, Dong-Liang Lv, Yu Li, Jian-Hong Hu

**Affiliations:** Key Laboratory of Animal Genetics, Breeding and Reproduction of Shaanxi Province, College of Animal Science and Technology, Northwest A&F University, Yangling 712100, China

## Abstract

Spermatogonial stem cells (SSCs) are the only adult stem cells that pass genes to the next generation and can be used in assisted reproductive technology and stem cell therapy. SSC cryopreservation is an important method for the preservation of immature male fertility. However, freezing increases the production of intracellular reactive oxygen species (ROS) and causes oxidative damage to SSCs. The aim of this study was to investigate the effect of melatonin on goat SSCs during cryopreservation and to explore its protective mechanism. We obtained SSCs from dairy goat testes by two-step enzymatic digestion and differential plating. The SSCs were cryopreserved with freezing media containing different melatonin concentrations. The results showed that 10^−6^ M of melatonin increased significantly the viability, total antioxidant capacity (T-AOC), and mitochondrial membrane potential of frozen-thawed SSCs, while it reduced significantly the ROS level and malondialdehyde (MDA) content (*P* < 0.05). Further analysis was performed by western blotting, flow cytometry, and transmission electron microscopy (TEM). Melatonin improved significantly the enzyme activity and protein expression of superoxide dismutase (SOD), catalase (CAT), and glutathione peroxidase (GSH-Px) (*P* < 0.05), thereby activating the antioxidant defense system of SSCs. Furthermore, melatonin inhibited significantly the expression of proapoptotic protein (Bax) and increased the expression of antiapoptotic proteins (Bcl-2 and Bcl-XL) (*P* < 0.05). The mitochondrial apoptosis pathway analysis showed that the addition of melatonin reduced significantly the mitochondrial swelling and vacuolation, and inhibited the release of cytochrome C from mitochondria into the cytoplasm, thereby preventing the activation of caspase-3 (*P* < 0.05) and inhibiting SSC apoptosis. In addition, melatonin reduced significantly the autophagosome formation and regulated the expression of autophagy-related proteins (LC3-I, LC3-II, P62, Beclin1, and ATG7) (*P* < 0.05), thereby reversing the freeze-induced excessive autophagy. In summary, melatonin protected goat SSCs during cryopreservation via antioxidant, antiapoptotic, and autophagic regulation.

## 1. Introduction

Spermatogonial stem cells (SSCs), which are types of primitive spermatogonia in the male reproductive system, are located in the basement membrane of the seminiferous tubules [1, 2]. SSCs both self-renew to keep the stability of the stem cell pool and differentiate to contribute to spermatogenesis, thereby maintaining the homeostasis of testicular cells [3]. As adult stem cells that produce spermatocytes, SSCs transmit genetic information to the next generation [4, 5]. Studies have shown that SSCs can be cultured and passaged in vitro, and differentiate into sperm after transplantation [6]. Therefore, the study of SSCs is significant in terms of understanding the male germ cell differentiation mechanism, and has clinical application value in the treatment of male infertility [7, 8]. However, the number of SSCs is low compared to other testicular cells, as, for example, these account for only 0.02–0.03% of the mouse testicular cells [9]. Therefore, the isolation, purification, identification, cultivation, preservation, and transplantation of SSCs have been the focus of recent research in biology and medicine [10, 11].

The cryopreservation of SSCs is an important method for preserving the fertility of high-quality immature dairy goats [8]. Freezing allows SSCs to be preserved for a long time, all the while preserving their biological characteristics, while after thawing they can be used in clinical settings [12]. However, during the freeze-thaw process, osmotic pressure, ice crystals, and reactive oxygen species (ROS) damage the structure and function of SSCs [13, 14]. The freezing process changes drastically the surroundings of SSCs and increases the production of intracellular ROS [15]. Unfortunately, the accumulation of excess ROS causes oxidative stress in cells, which reduces the viability of frozen-thawed SSCs and even leads to apoptosis [16]. Therefore, the oxidative damage that results from SSC cryopreservation is a problem that needs to be solved urgently.

Melatonin, an indoleamine hormone synthesized by the pineal gland, is generally regarded as an effective antioxidant and free radical scavenger in various cells [17, 18]. Melatonin has beneficial effects in the context of clinical medicine and cell cultures in vitro, including antioxidation, antiapoptosis, anti-inflammation, and autophagy and circadian rhythm regulation [19, 20]. Many studies have shown that melatonin protects cells against oxidative stress, and its ability to scavenge free radicals is better than that of vitamin E [21, 22]. Therefore, melatonin is used as an effective cell protective agent and a potential disease prevention agent [23]. Melatonin protects cells against oxidative stress in multiple ways, and the main mechanism of its reaction with free radicals consists of electron transfer and hydrogen transfer [24]. Furthermore, Rodriguez et al. [25] showed that melatonin regulates the gene expression and enzyme activity of the antioxidant enzymes glutathione peroxidase (GSH-Px), superoxide dismutase (SOD), and catalase (CAT). Li et al. [26] showed that melatonin ameliorated the busulfan-induced apoptosis in the SSCs of mice, which was caused by high concentrations of ROS and p53, by promoting the expression of manganese SOD and sirtuin type 1. Therefore, we wondered whether melatonin has a beneficial effect on goat SSCs during cryopreservation.

The purpose of this study was to investigate the effect of melatonin on goat SSCs during cryopreservation and to explore its protective mechanism. The cell viability, ROS level, malondialdehyde (MDA) content, total antioxidant capacity (T-AOC), mitochondrial membrane potential, and antioxidant enzyme activity of SSCs were evaluated after freezing-thawing to determine the most effective melatonin concentration. In addition, we detected the oxidation, apoptosis, and autophagy indicators by western blotting, flow cytometry, and transmission electron microscopy (TEM), to explore the melatonin protection mechanism of goat SSCs during cryopreservation.

## 2. Materials and Methods

**Figure 1:**
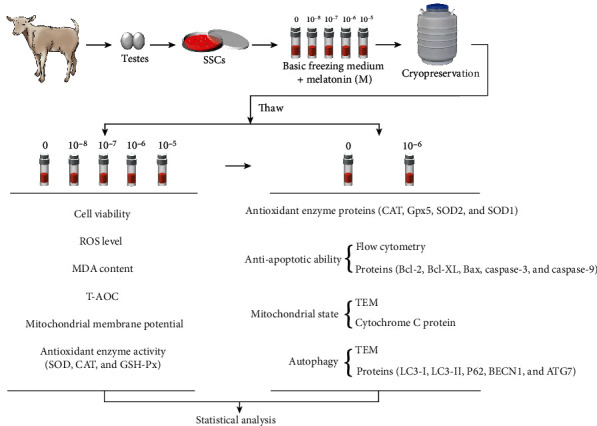
The schematic illustration representing the whole experiment. SSCs: spermatogonial stem cells; ROS: reactive oxygen species; MDA: malondialdehyde; T-AOC: total antioxidant capacity; SOD: superoxide dismutase; CAT: catalase; GSH-Px: glutathione peroxidase; TEM: transmission electron microscopy.

The schematic illustration representing the whole experiment is shown in Figure 1.

### 2.1. Animals and Testis Collection

The animal experiment procedures of this study were approved by the Institutional Animal Care and Use Committee of the Northwest A&F University, Shaanxi, China (NWAFU-201937024, March 2019). Testis samples were obtained from 2–2.5-month-old male dairy goats that belonged to the Shaanxi Organic Dairy Goat Breeding Co., Ltd. These dairy goats were placed in a comfortable environment and were provided with balanced food and clean water. Clinical operations including anesthesia, disinfection, ligation, excision, and suture were performed by professionals in a sterile environment, and to ensure the health of the goats. Goat testes were collected under aseptic conditions and rinsed with phosphate-buffered saline (PBS; SH30256.01; Hyclone, Logan, UT, USA) containing 5% penicillin-streptomycin (SV30010; Hyclone). The samples were then stored in test tubes with PBS and placed in an icebox to facilitate their transportation to the laboratory. All experiments in this study were conducted with three biological replicates.

### 2.2. Isolation and Purification of Goat SSCs

The experimental protocol for sample preparation is referred to Heidari et al. [27] with minor modifications. The tunica albuginea was removed from the testes. Then, the shredded testis tissues were rinsed with Dulbecco's phosphate-buffered saline (DPBS; SH30028.02; Hyclone). A solution containing 1 mg/mL collagenase type IV (V900893; Sigma, St. Louis, MO, USA), 1 mg/mL hyaluronidase (H3506; Sigma), and 25 *μ*g/mL DNase I (DN25; Sigma) was added to the samples and shaken at 37°C for 25 min. The samples were centrifuged and the supernatant was discarded; then, they were suspended in DPBS and the seminiferous tubules were collected. Next, a red blood cell lysis buffer (R1010; Solarbio, Beijing, China) was added to the samples, followed by the addition of 0.25% trypsin (T1426; Sigma). The samples were examined microscopically. After the cells were completely dispersed, Dulbecco's modified Eagle medium (DMEM; SH30243.01; Hyclone) solution containing 10% fetal bovine serum (FBS; 10270106; Gibco, Carlsbad, CA, USA) was added to terminate the reaction. The dispersed cells were filtered through a 40 *μ*m strainer (352340; Falcon, USA). The filtrate was centrifuged, and the pellet was suspended in a DMEM solution containing 2% FBS. The cells were preserved in a 37°C incubator with 5% CO_2_ (Thermo Fisher). The testicular cells were subjected to differential plating in order to purify the goat SSCs. The separated cells were placed in a 60 mm culture dish coated with 0.2% gelatin (R00501; Leagene Biotech Co. Ltd., Beijing, China). After 2 h of incubation, the nonadherent cells were transferred into a new culture dish and incubated at 37°C for 4 h. Then, the nonadherent cells were pipetted into a new culture dish and incubated overnight. The next day, the sample in the culture dish was collected gently using a pipette. Cells were plated at a density of 1 to 2 × 10^5^ cells/well on 12-well plates (3513; Corning). SSCs were cultured in DMEM supplemented with 2% FBS, 10 ng/mL glial cell-derived neurotrophic factor (GDNF; P39905; R&D Systems, USA), 2 mM L-glutamine (SH30034.01; Hyclone), and 2% penicillin-streptomycin in a humidified atmosphere at 37°C with 5% CO_2_. The medium was replaced every 2 days.

### 2.3. Immunofluorescence Staining

In this study, we used promyelocytic leukemia zinc finger (PLZF) and Thy-1 cell surface antigen (THY1) as specific markers of goat SSCs for immunofluorescence staining. Briefly, the cells were pipetted into a 96-well plate (3599; Corning) precoated with poly-L-lysine (P2100; Solarbio), with approximately 5 × 10^3^ cells per well. Then, the cells were fixed in 4% paraformaldehyde (PFA; P1110; Solarbio) for 15 min and then permeabilized by the addition of 0.25% Triton X-100 (T8200; Solarbio). Thereafter, 10% of goat serum was added for blocking. A diluted primary antibody (1 : 200; bs-5971R and bs-0778R; Bioss, Beijing, China) was added and incubated overnight at 4°C. After rinsing with PBST, a diluted fluorescent secondary antibody (1 : 200; bs-0295G-Cy3 and bs-0295G-FITC; Bioss) was added and incubated for 1 h at room temperature. After incubation, the cells were rinsed three times with PBST. We added 4′,6-diamidino-2-phenylindole dihydrochloride (DAPI; C0060; Solarbio), and the cells were rinsed after 5 min of incubation. The cells were observed under a fluorescence microscope (Nikon, Tokyo, Japan).

### 2.4. SSC Cryopreservation

The basic freezing medium consisted of *α*-minimum Eagle's medium (*α*-MEM; 12571071; Gibco), 10% dimethyl sulfoxide (DMSO; D8371; Solarbio), 10% FBS, 10 mM HEPES (H1095; Solarbio), 2 mM L-glutamine, and 50 *μ*g/mL penicillin-streptomycin. We used *α*-MEM to dissolve melatonin (M8600; Solarbio), and stored it in a refrigerator at -20°C. The melatonin solution was taken out when used, and mixed with other reagents in proportion. The freezing medium in the treatment groups consisted of melatonin, which was added to the basic medium at final concentrations of 10^−8^, 10^−7^, 10^−6^, and 10^−5^ M.

The goat SSCs were suspended in a freezing medium at a concentration of 1 to 2 × 10^6^ cells per mL and aliquoted into 2 mL cell cryovials (430659; Corning, USA). The cryovials were packed in a freezing container and placed in a -80°C refrigerator for 24 h. After being incubated overnight, the cryovials were immersed in liquid nitrogen and stored for one month.

### 2.5. Cell Viability Analysis

We analyzed the vitality of frozen-thawed goat SSCs. After being cryopreserved, the SSCs were incubated in a 37°C water bath in order to thaw. Then, 0.4% trypan blue solution (C0040; Solarbio) was added for staining, and the dead cells were stained blue. A blood cell counter was used to count the number of dead and living cells, and cell viability was calculated. In addition, we also used the MTT Cell Proliferation and Cytotoxicity Assay Kit (M1020; Solarbio) to detect cell viability after 24 h of culture. According to the manufacturer's instructions, 90 *μ*L cell solution (5 × 10^3^ cells) was mixed with 10 *μ*L 3-(4,5-dimethylthiazol-2-yl)-2,5-diphenyl tetrazolium bromide (MTT) solution per well. After 4 h of incubation, the supernatant was removed, and the DMSO was added to dissolve the formazan. After shaking for 10 min, the optical density (OD) at 490 nm was measured.

### 2.6. Intracellular ROS Measurement

The intracellular ROS levels were measured using the fluorescent probe 2,7-dichlorodihydrofluorescein diacetate (DCFH-DA). We used a Reactive Oxygen Species Assay Kit (CA1410; Solarbio) and followed the manufacturer's instructions. The SSCs (10^6^ cells per sample) were incubated in a serum-free medium containing 10 *μ*M DCHF-DA at 37°C for 20 min and then washed three times with PBS. The values were detected using a microplate reader (Synergy H1; BioTek, USA) at a 488 nm excitation wavelength and a 525 nm emission wavelength.

### 2.7. MDA Content and T-AOC Determination

To determine the lipid peroxidation levels, MDA, the product of cellular lipid oxidation, was evaluated. The samples (10^6^ cells per sample) were mixed with thiobarbituric acid (TBA; BC0020; Solarbio) to form a brown-red complex. The absorbance was measured at 532 nm using a spectrophotometer. The T-AOC of SSCs (10^6^ cells per sample) was measured using the Total Antioxidant Capacity Assay Kit (BC1310; Solarbio) based on the ferric reducing antioxidant power (FRAP) assay. The samples were detected at 593 nm, and the T-AOC was calculated according to the standard curve.

### 2.8. Mitochondrial Membrane Potential Detection

Changes in the mitochondrial membrane potential reflect the early apoptosis of cells. The JC-1 Mitochondrial Membrane Potential Detection Kit (M8650; Solarbio) was used to detect the mitochondrial membrane potential of the SSCs according to the manufacturer's instructions. Briefly, the cells (10^6^ cells per sample) were mixed with JC-1 dye and incubated at 37°C for 20 min. Normal cells emit red fluorescence, while cells with a decreased mitochondrial membrane potential emit green fluorescence. The mitochondrial membrane potential was determined by calculating the red to green fluorescence ratio.

### 2.9. Antioxidant Enzyme Activity Assay

SOD, CAT, and GSH-Px are key antioxidant enzymes in SSCs. The SOD activity was measured using a xanthine and xanthine oxidase reaction system, while the CAT activity was measured using the decomposition of hydrogen peroxide. The detection was performed using a spectrophotometry kit (10^6^ cells per sample), according to the manufacturer's instructions (BC0175 and BC0200; Solarbio). GSH-Px catalyzes the conversion of reduced glutathione (GSH) to oxidized glutathione (GSSG). The GSH and GSSG contents in the cells were detected using a GSH Content Detection Kit (BC1170; Solarbio) and a GSSG Content Detection Kit (BC1180; Solarbio), respectively. The GSH-Px activity was indirectly determined by the GSH/GSSG ratio.

### 2.10. Western Blotting

We used western blotting to detect the protein expression of SSCs in the control group and the melatonin treatment group (10^−6^ M). The SSCs were collected by centrifugation and lyzed using a radioimmunoprecipitation (RIPA) lysis buffer (P0013B; Beyotime, Shanghai, China) containing the protease inhibitor phenylmethylsulfonyl fluoride (PMSF; ST506, Beyotime). The loading buffer was added to the protein samples according to the protein concentration and boiled for 10 min. The SSC proteins were then separated by 12% sodium dodecyl sulfate-polyacrylamide gel electrophoresis (SDS-PAGE; AR0138; Boster, Wuhan, China). Next, the imprint was electrotransferred to a polyvinylidene fluoride (PVDF) membrane (AR0136; Boster). The PVDF membrane was blocked for 1 h and washed with Tris-buffered saline containing 0.1% Tween-20 (TBST; T8220; Solarbio). The membrane was incubated in the diluted primary antibody at 4°C overnight. The primary antibodies used in this study included intracellular antioxidant enzyme protein antibodies anti-CAT (1 : 1000; 66765-1-Ig; Proteintech, Chicago, IL, USA), anti-Gpx5 (1 : 1000; 18731-1-AP; Proteintech), anti-SOD2 (1 : 1000; 24127-1-AP; Proteintech), and anti-SOD1 (1 : 1000; 10269-1-AP; Proteintech), apoptosis-related protein antibodies anti-Bcl-2 (1 : 1000; ab32124; Abcam, Cambridge, MA, USA), anti-Bcl-XL (1 : 1000; ab32370; Abcam), anti-Bax (1 : 1000; 2772; Cell Signaling Technology, Danvers, MA, USA), anti-caspase-3 (1 : 1000; ab13847; Abcam), and anti-caspase-9 (1 : 1000; ab32539; Abcam), and autophagy-related protein antibodies anti-LC3B (1 : 2000; ab192890; Abcam), anti-P62 (1 : 1000; 18420-1-AP; Proteintech), anti-Beclin1 (1 : 1000; 66665-1-Ig; Proteintech), anti-ATG7 (1 : 1000; ab223380; Abcam), and *β*-tubulin (1 : 1000; KM9003T; Sungene Biotech, Tianjin, China). After being washed three times with TBST, the membrane was incubated in the diluted secondary antibody (horseradish peroxidase-conjugated goat anti-rabbit IgG or goat anti-mouse IgG) in a shaker for 1 h. Protein bands were visualized on a Gel Doc XR System (Bio-Rad, Berkeley, CA, USA) using an ECL western blot kit (32106; Thermo Fisher). In addition, we extracted the mitochondrial proteins of the SSCs in the control and melatonin treatment group (10^−6^ M) and performed western blot analysis using anti-cytochrome C (1 : 1000; 10993-1-AP; Proteintech) and anti-COX IV (1 : 1000; 11242-1-AP; Proteintech) as described above.

### 2.11. Flow Cytometry

Annexin V-FITC/PI double staining (G003-1-2; Nanjing Jiancheng Bioengineering Institute, Jiangsu, China) was used to stain the cells, and flow cytometry was used to detect cell apoptosis. Annexin V is a sensitive indicator of early cell apoptosis. Propidium iodide (PI) is a nucleic acid dye that stains dead cells and cells in the metaphase and advanced stages of apoptosis. The SSCs were collected by centrifugation and suspended in a 1× binding buffer to adjust the cell concentration to 1 × 10^6^/mL. Annexin V-FITC (5 *μ*L) and PI (10 *μ*L) were added to the tubes and mixed gently. After incubation, the samples were placed in an ice bath in the dark and then analyzed by a flow cytometry machine (Beckman, CA, USA).

### 2.12. Transmission Electron Microscopy (TEM)

TEM was used to characterize the morphology and structure of SSC mitochondria and autophagosomes. Briefly, the frozen-thawed SSCs were washed with PBS and centrifuged to remove the supernatant. The cells were fixed with 2.5% glutaraldehyde (P1126; Solarbio) for 4–6 h and then fixed with 1% osmium tetroxide for 2–4 h. Next, the samples were dehydrated in graded alcohol and embedded in Epon. The prepared specimens were ultrathin sectioned and added to the copper grid. TEM (Hitachi, Japan) was used to visualize changes in the ultrastructure of SSCs, with 15 or 12 cells per group.

### 2.13. Statistical Analysis

All experiments in this study were repeated three times. All statistical analyses were performed using IBM SPSS Statistics for Windows version 21.0 (IBM Corp., Armonk, NY, USA). Duncan's new multiple range test was used to evaluate the differences. The values were expressed as the mean ± standard deviation (SD). *P* < 0.05 was considered statistically significant.

## 3. Results

### 3.1. Identification of Goat SSCs by Morphology and Immunofluorescence Analysis

**Figure 2:**
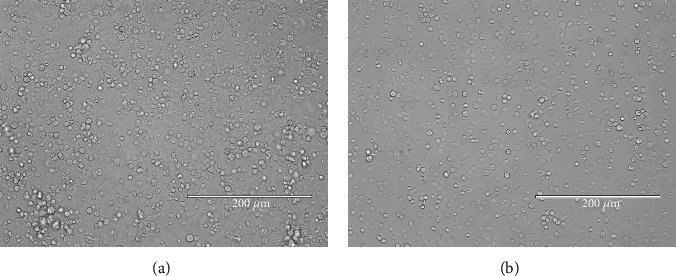
Isolation and purification of goat spermatogonial stem cells (SSCs). (a) Testicular cells isolated from dairy goat testes by two-step enzymatic digestion. (b) Goat SSCs after differential plating. Scale bar = 200 *μ*m.

**Figure 3:**
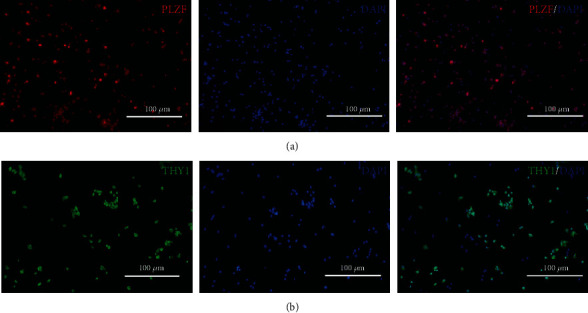
Immunofluorescence staining of promyelocytic leukemia zinc finger (PLZF) and Thy-1 cell surface antigen (THY1) on goat spermatogonial stem cells (SSCs). (a) PLZF (red). (b) THY1 (green). Nuclear staining is indicated by 4′,6-diamidino-2-phenylindole dihydrochloride (DAPI) (blue). Scale bar = 100 *μ*m.

The unpurified testicular cell suspension contained multiple cells (Figure 2(a)). After differential plating, the collected cells were uniform in size and round in shape (Figure 2(b)). These purified cells conformed to the SSC morphological characteristics. Immunofluorescence staining of SSC markers PLZF and THY1 was used to identify cells. As is shown in Figure 3, cells stained red (PLZF+) or green (THY1+) were considered to be SSCs.

### 3.2. Effects of Different Melatonin Concentrations on SSC Viability during Cryopreservation

**Figure 4:**
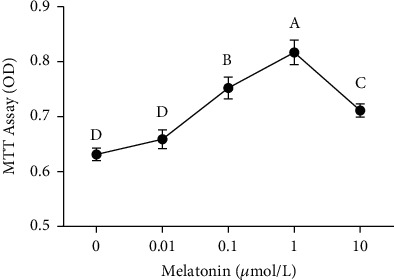
Effects of different melatonin concentrations on spermatogonial stem cell (SSC) viability during cryopreservation were evaluated by MTT assay. Different superscript letters (A–D) indicate significant differences (*P* < 0.05). Values are expressed as the means ± SD (*n* = 3).

Table 1 shows the viability of frozen-thawed SSCs treated with different melatonin concentrations. The results showed that, compared with the control group, the melatonin treatment improved significantly the SSC viability after thawing (*P* < 0.05). At a melatonin concentration of 1 *μ*M, the SSC viability reached 68.99%, which was the highest among all treatment groups (*P* < 0.05).  Figure 4 shows the results of the MTT assay and also verified that 1 *μ*M melatonin had the best protective effect.

### 3.3. Effects of Different Melatonin Concentrations on the ROS Levels in SSCs during Cryopreservation

**Figure 5:**
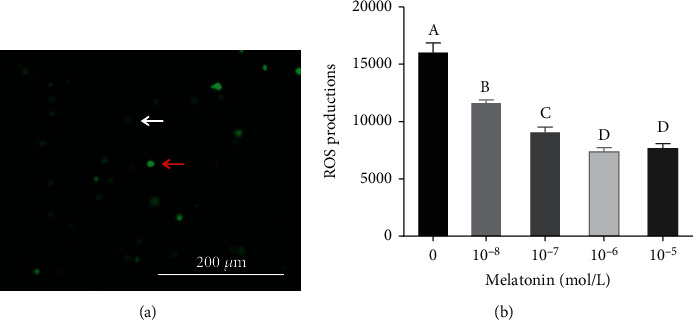
Effects of different melatonin concentrations on the reactive oxygen species (ROS) level of spermatogonial stem cells (SSCs) during cryopreservation. (a) Intracellular ROS fluorescence. The red and white arrows indicate higher and lower ROS levels, respectively. (b) Intracellular ROS content in different groups. Different superscript letters (A–D) indicate significant differences (*P* < 0.05). Values are expressed as the means ± SD (*n* = 3). Scale bar = 200 *μ*m.

As is shown in Figure 5, the intracellular ROS levels were significantly lower in all melatonin treatment groups compared to that in the control group (*P* < 0.05). In particular, the group supplemented with 10^−6^ M and 10^−5^ M of melatonin had significantly lower intracellular ROS than the other groups (*P* < 0.05); however, the difference between the two groups was not significant (*P* > 0.05).

### 3.4. Effects of Different Melatonin Concentrations on the MDA Content and T-AOC of SSCs during Cryopreservation

**Figure 6:**
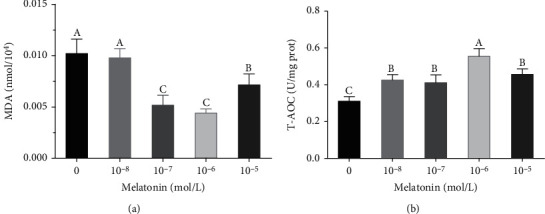
Effects of different melatonin concentrations on the malondialdehyde (MDA) content and total antioxidant capacity (T-AOC) of spermatogonial stem cells (SSCs) during cryopreservation. (a) MDA content in different groups. (b) T-AOC in different groups. Different superscript letters (A–C) indicate significant differences (*P* < 0.05). Values are expressed as the means ± SD (*n* = 3).

The results of the MDA content determination (Figure 6(a)) showed that the addition of 10^−7^, 10^−6^, and 10^−5^ M of melatonin to the freezing medium significantly reduced the MDA production in SSCs (*P* < 0.05). The lowest MDA content was observed in the 10^−6^ M melatonin group; however, there was no significant difference between this and the 10^−7^ M melatonin group (*P* > 0.05). In terms of antioxidant capacity, the SSCs in the 10^−6^ M melatonin treatment group had the highest T-AOC among all groups (*P* < 0.05, Figure 6(b)).

### 3.5. Effects of Different Melatonin Concentrations on the Mitochondrial Membrane Potential of SSCs during Cryopreservation

**Figure 7:**
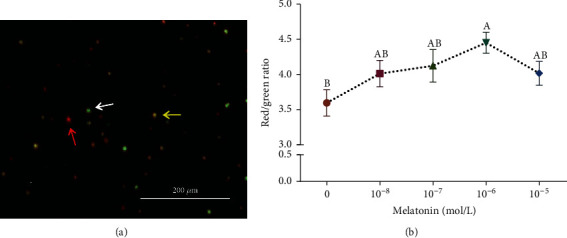
Effects of different melatonin concentrations on the mitochondrial membrane potential of spermatogonial stem cells (SSCs) during cryopreservation. (a) JC-1 staining fluorescence. The red arrow indicates a normal mitochondrial membrane potential, while the yellow and white arrows indicate decreased mitochondrial membrane potential. (b) Mitochondrial membrane potential in different groups. Different superscript letters (A, B) indicate significant differences (*P* < 0.05). Values are expressed as the means ± SD (*n* = 3).

The mitochondrial membrane potential of SSCs in each group after freezing-thawing is shown in Figure 7. The melatonin treatments increased significantly the mitochondrial membrane potential of SSCs (*P* < 0.05) compared with the control group. The red/green fluorescence ratio reached its maximum level under the 10^−6^ M melatonin concentration, thereby indicating the highest mitochondrial membrane potential under the aforementioned melatonin concentration (*P* < 0.05).

### 3.6. Effects of Different Melatonin Concentrations on the Antioxidant Enzyme Activity of SSCs during Cryopreservation

**Figure 8:**
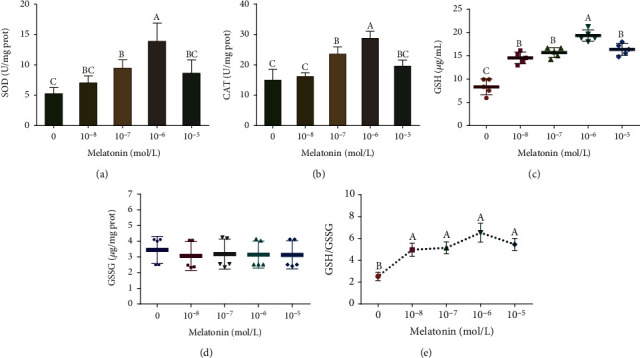
Effects of different melatonin concentrations on the antioxidant enzyme activity of spermatogonial stem cells (SSCs) during cryopreservation. (a) Superoxide dismutase (SOD) activity. (b) Catalase (CAT) activity. (c) Reduced glutathione (GSH) activity. (d) Oxidized glutathione (GSSG) activity. (e) GSH/GSSG ratio, which is an indirect indicator of glutathione peroxidase (GSH-Px) activity. Different superscript letters (A–C) indicate significant differences (*P* < 0.05). Values are expressed as the means ± SD (*n* = 3).

In order to explore the ability of antioxidant enzymes in SSCs, the SOD, CAT, and GSH-Px activities were assayed. As is shown in Figure 8, the freezing medium containing melatonin resulted in higher SOD and CAT activities compared to the corresponding enzyme activities in the control group (*P* < 0.05). The 10^−6^ M melatonin treatment group had the highest enzyme activity. The GSH/GSSG ratio reflects the redox state of the cell and is an indirect indicator of GSH-Px activity. The results of this study showed that the GSH/GSSG value of the melatonin treatment groups was significantly higher than that of the control group (*P* < 0.05), but the difference between the treatment groups was not significant (*P* > 0.05).

### 3.7. Effects of Melatonin on the Expression of Antioxidant Enzyme Proteins in SSCs during Cryopreservation

**Figure 9:**
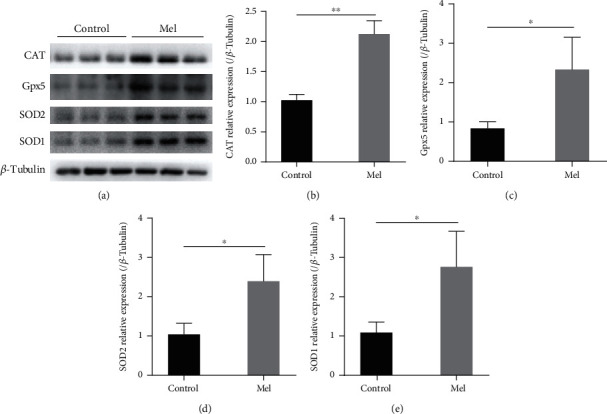
Antioxidant enzyme proteins in frozen-thawed goat spermatogonial stem cells (SSCs) detected by western blotting. (a) Western blot image of catalase (CAT), glutathione peroxidase 5 (Gpx5), superoxide dismutase 2 (SOD2), and SOD1. (b–e) Relative protein expression in the control and 10^−6^ M melatonin treatment group. The superscript ∗ indicates a significant difference at *P* < 0.05, and the superscript ∗∗ indicates a significant difference at *P* < 0.01. The values are expressed as the means ± SD (*n* = 3). Mel: 10^−6^ M melatonin.

The expression of antioxidant enzyme proteins in SSCs in the 10^−6^ M melatonin treatment and control groups was detected by western blotting. As is shown in Figure 9, the addition of 10^−6^ M melatonin during cryopreservation increased significantly the CAT, Gpx5, SOD2, and SOD1 expression in goat SSCs (*P* < 0.05) compared to the control group. These analyses indicate that melatonin may protect SSCs from oxidative damage by promoting the activity and expression of intracellular antioxidant enzymes.

### 3.8. Effects of Melatonin on the Antiapoptotic Ability of SSCs during Cryopreservation

**Figure 10:**
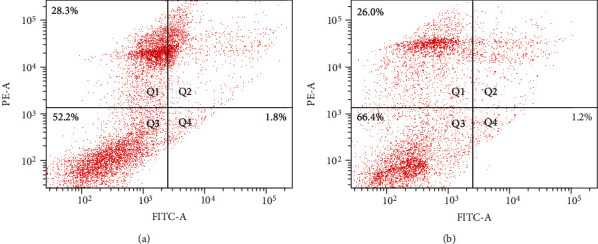
Flow cytometry analysis for the detection of spermatogonial stem cell (SSC) apoptosis. (a) Control group. (b) 10^−6^ M melatonin treatment group.

Figure 10 shows the apoptosis of SSCs detected by flow cytometry with Annexin V-FITC/PI staining. The results showed that the SSCs in the control group consisted of 52.2% viable cells, 1.8% early apoptotic cells, and 28.3% nonviable apoptotic cells (Figure 10(a)). The SSCs in the 10^−6^ M melatonin treatment group consisted of 66.4% viable cells, 1.2% early apoptotic cells, and 26.0% nonviable apoptotic cells (Figure 10(b)). This indicated that melatonin increased visibly the number of surviving cells after the freeze-thaw process and reduced apoptosis effectively.

**Figure 11:**
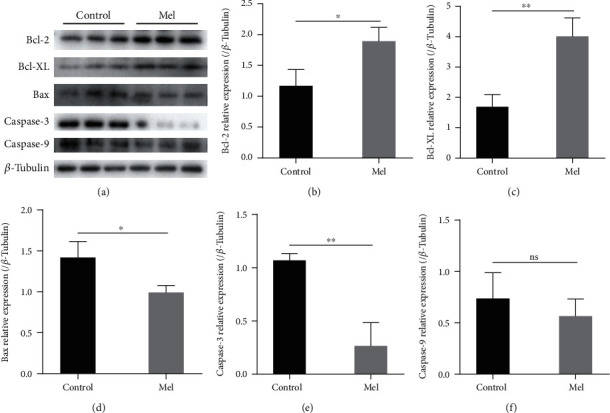
Apoptosis-related proteins in frozen-thawed goat spermatogonial stem cells (SSCs), which were detected by western blotting. (a) Western blot image of Bcl-2, Bcl-XL, Bax, caspase-3, and caspase-9. (b–f) Relative protein expression in the control and 10^−6^ M melatonin treatment group. The superscript ∗ indicates a significant difference at *P* < 0.05, and the superscript ∗∗ indicates a significant difference at *P* < 0.01. The superscript ns indicates no significant difference. Values are expressed as the means ± SD (*n* = 3). Mel: 10^−6^ M melatonin.

The expression levels of apoptosis-related proteins were determined by western blotting (Figure 11). The addition of melatonin resulted in a significant increase in the expression of the antiapoptotic proteins Bcl-2 and Bcl-XL in SSCs during cryopreservation (*P* < 0.05). In contrast, the addition of melatonin resulted in a significant decrease in the expression of the proapoptotic protein Bax (*P* < 0.05). The full name of caspase is cysteinyl aspartate specific proteinase, which is closely related to eukaryotic cell apoptosis. The expression of the caspase-3 protein, a key executor of apoptosis, was significantly lower in the 10^−6^ M melatonin treatment group than in the control group (*P* < 0.05). However, the expression of apoptosis initiator caspase-9 was not significantly different between the two groups (*P* > 0.05).

### 3.9. Effects of Melatonin on the Mitochondrial State of SSCs during Cryopreservation

**Figure 12:**
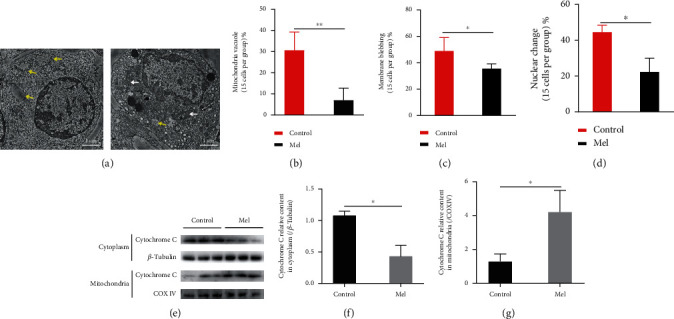
Mitochondrial status of frozen-thawed goat spermatogonial stem cells (SSCs). (a) The mitochondrial swelling and vacuolation in goat SSCs were characterized by transmission electron microscopy (TEM). The yellow arrows indicate swollen and vacuolated mitochondria, and the white arrow indicates normal mitochondria. (b) Statistical analysis results of mitochondrial swelling and vacuolation. (c) Membrane blebbing. (d) Nuclear change. (e) Western blot image of cytochrome C in the cytoplasm and mitochondria. (f, g) Relative protein expression in the control and 10^−6^ M melatonin treatment group. The superscript ∗ indicates a significant difference at *P* < 0.05, and the superscript ∗∗ indicates a significant difference at *P* < 0.01. Values are expressed as the means ± SD (*n* = 3). Mel: 10^−6^ M melatonin. Scale bar = 1 *μ*m.

TEM was used to characterize mitochondrial swelling and vacuolation in goat SSCs (Figure 12(a)). Fifteen cells were randomly selected from each group for statistical analysis. The results showed that the mitochondrial swelling and vacuolation in the 10^−6^ M melatonin treatment group was significantly lower than that in the control group (*P* < 0.05, Figure 12(b)). And the addition of melatonin resulted in a significant reduction in the percentage of cells with membrane blebbing and nuclear change (*P* < 0.05, Figures 12(c) and 12(d)).

The western blot analysis of cytochrome C protein (Figure 12(e)) showed that the cytochrome C content in the cytoplasm of control group cells was significantly higher than that in the melatonin treatment group cells (*P* < 0.05, Figure 12(f)). In contrast, the cytochrome C content in the mitochondria of the control group was significantly lower than that in the mitochondria of the melatonin treatment group (*P* < 0.05, Figure 12(g)). Cytochrome C is transferred from the mitochondria to the cytoplasm during a freezing injury, which activates the downstream apoptosis pathway and, ultimately, leads to apoptosis. The addition of melatonin alleviated the mitochondrial pathway of SSC apoptosis during cryopreservation.

### 3.10. Effects of Melatonin on SSC Autophagy during Cryopreservation

**Figure 13:**
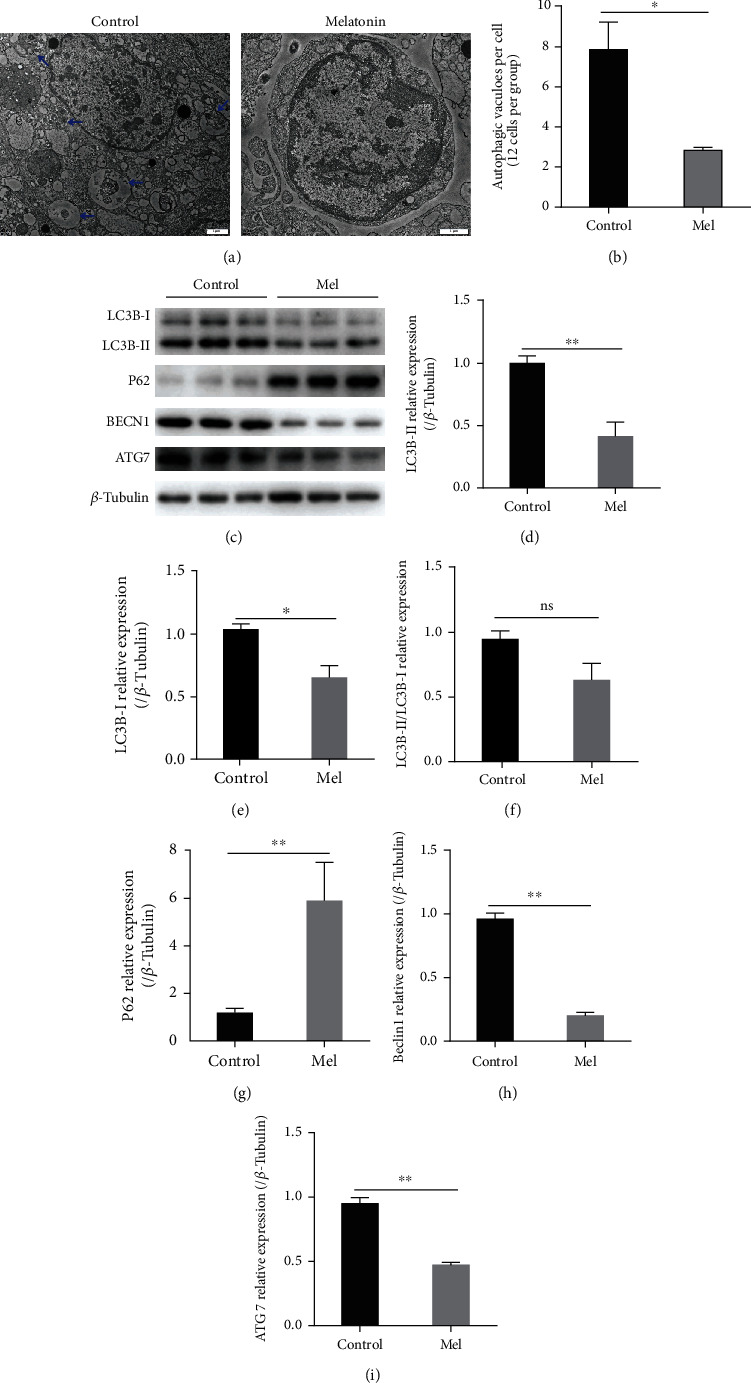
Melatonin effects on the autophagy of spermatogonial stem cells (SSCs) during cryopreservation. (a) Autophagosomes in goat SSCs were characterized by transmission electron microscopy (TEM). (b) The number of autophagosomes. (c) Western blot image of LC3-I, LC3-II, P62, BECN1, and ATG7. (d–i) Relative protein expression in the control group and 10^−6^ M melatonin treatment group. The superscript ∗ indicates a significant difference at *P* < 0.05, and the superscript ∗∗ indicates a significant difference at *P* < 0.01. The superscript ns indicates no significant difference. Values are expressed as the means ± SD (*n* = 3). Mel: 10^−6^ M melatonin. Scale bar = 1 *μ*m.

TEM analysis was performed to observe the ultrastructural changes of SSCs and to count the autophagosomes present (Figure 13(a)). The results showed that the number of autophagosomes produced by SSCs in the 10^−6^ M melatonin treatment group was significantly lower than that in the control group (*P* < 0.05, Figure 13(b)).

The relative expression of autophagy-related proteins was detected by western blotting (Figures 13(c)–13(i)). The melatonin treatment reduced significantly the expression of the LC3-I and LC3-II autophagy markers in SSCs (*P* < 0.05), while it increased significantly the expression of the P62 protein (*P* < 0.05). The autophagy flux of SSCs was dysregulated during cryopreservation and the cells suffered excessive autophagy; however, melatonin suppressed this effect. Beclin1 is involved in the feedback regulation of autophagy and apoptosis and can interact with Bcl-2. The western blot results showed that the expression of the Beclin1 protein in the melatonin treatment group was significantly lower than that in the control group (*P* < 0.05). In addition, the expression of autophagosome formation-related protein ATG7 showed the same trend (*P* < 0.05). The analysis revealed that the addition of melatonin to the freezing medium reduced the excessive autophagy of SSCs caused by cold stress.

## 4. Discussion

SSCs are the only adult stem cells that pass genes to offspring and have been used in the treatment of male reproductive system diseases and animal genome editing [5]. This study investigated for the first time the effect of melatonin on goat SSCs during cryopreservation and explored its protective mechanisms in terms of oxidation, apoptosis, and autophagy. The results showed that melatonin increased significantly the viability of frozen-thawed SSCs, the T-AOC, mitochondrial membrane potential, and the activities of antioxidant enzymes SOD, CAT, and GSH-Px, while it reduced significantly the ROS level and the MDA content. The optimum melatonin concentration was 10^−6^ M. Flow cytometry, western blotting, and TEM were used to analyze the degree of cell apoptosis, protein expression, and ultrastructure. These results indicate that melatonin protected goat SSCs during cryopreservation by improving their antioxidant capacity, inhibiting the mitochondrial pathway of apoptosis, and regulating autophagy.

As SSCs are rare in the testes, it is crucial to isolate and purify SSCs from goat testes before cryopreservation [28]. In this work, we obtained SSCs from goat testes by two-step enzymatic digestion and enriched the cells by differential plating. The SSCs were identified by the immunofluorescence staining of PLZF and THY1. PLZF and THY1 have been shown to be SSC markers [29, 30]. The results showed that the SSCs examined in this study were positive for PLZF and THY1. Then, the SSCs were added to the self-made freezing medium for cryopreservation.

The cryopreservation procedure increases the production of ROS in SSCs [15]. The accumulation of excess ROS causes oxidative stress and impairs the cell vitality, structure, and function [31]. Elevated ROS levels also induce mitochondrial permeability transition, and cytochrome C is released into the cytoplasm, thereby promoting apoptosis [32]. In addition, the abnormal physiological environment induces excessive autophagy to reutilize its own constituents for energy, thereby causing cell death [33]. Therefore, the addition of effective reagents in the SSC freezing medium to regulate oxidation, apoptosis, and autophagy is important for SSC cryopreservation.

Melatonin is a powerful antioxidant that can scavenge different types of free radicals in the cells and activate the antioxidant defense system [34]. Melatonin is considered to be a protector of cells and mitochondria and prevents the attenuation of the mitochondrial membrane potential and energy production, thereby reducing mitochondrial oxidative stress [35]. Melatonin supplementation reduces the production of ROS in cells and prevents mitochondrial apoptosis signaling via the specific targeting of Bcl2/Bax [36]. Tian et al. [19] showed that melatonin promotes the development of microinjected pronuclear mouse embryos in vitro through its antioxidant and antiapoptotic effects.

In this study, supplementing the freezing medium with melatonin improved significantly the quality of goat SSCs during cryopreservation. The optimal concentration of melatonin was 10^−6^ M, and the viability of frozen-thawed SSCs was 68.99%. Importantly, melatonin reduced significantly the accumulation of intracellular ROS and the production of the lipid peroxidation product MDA, improved the T-AOC of SSCs, and prevented effectively the oxidative damage of SSCs. Navid et al. [37] showed that the addition of 100 *μ*M of melatonin to the culture medium decreases significantly the production of ROS in SSCs, which is consistent with the results of our study. Mitochondria are the major site of adenosine triphosphate (ATP) production and the main site of intracellular ROS production [38]. The opening of mitochondrial permeability transition pores has been shown to induce the depolarization of the transmembrane potential; therefore, the mitochondrial membrane potential could very well reflect the mitochondrial state [39]. The results of the JC-1 test showed that melatonin prevented effectively the reduction of the mitochondrial membrane potential caused by freezing and oxidation. Pena et al. [40] and Liu et al. [41] also demonstrated the role of melatonin in preserving the mitochondrial membrane potential. The main antioxidant enzymes in the cell are SOD, CAT, and GSH-Px. The main SOD protein forms are SOD1 (Cu-ZnSOD, cytosolic) and SOD2 (MnSOD, mitochondrial), which dismutate the superoxide radical into hydrogen peroxide and oxygen, and then GSH-Px and CAT detoxify the hydrogen peroxide into water [42]. In the current study, melatonin increased significantly the enzyme activity and protein expression of SOD, CAT, and GSH-Px. This is consistent with the results of Mukherjee et al. [43] on hamster testes. Therefore, melatonin may preserve the stability of the cellular antioxidant defense system by directly scavenging free radicals and increasing the antioxidant enzyme activity and expression, thereby improving the effect of SSC cryopreservation.

The regulation of redox, apoptosis, and autophagy plays an important role in the physiological metabolism of cells. Studies have shown that melatonin reduces cell apoptosis and autophagosome formation by preserving the cell redox balance [44]. The Bax and Bcl-2 proteins regulate the cellular mitochondrial membrane permeability and are important pathways for apoptosis regulation [45]. In this study, western blotting was used to detect the expression of apoptosis-related proteins in SSCs. The results showed that the expression of the antiapoptotic proteins Bcl-2 and Bcl-XL in the melatonin treatment group increased significantly, while the expression of the proapoptotic protein Bax and the apoptosis pathway-related protein caspase-3 decreased significantly. This is consistent with the results of Xu et al. [46], who suggested that melatonin is involved in cell apoptosis and necrosis by modulating the Bcl-2/Bax balance. Moreover, the flow cytometry results showed that the number of apoptotic cells decreased and the number of surviving cells increased in the 10^−6^ M melatonin treatment group. Mitochondria regulate apoptosis through the intrinsic pathway. Cryopreservation and excessive ROS trigger mitochondrial swelling and vacuolation, and open the mitochondrial permeability transition pore. This causes mitochondria to release cytochrome C to the cytoplasm, which, along with deoxyadenosine triphosphate (dATP), binds to Apaf-1, which then binds to procaspase-9. Caspase-9 further activates caspase-3, thereby leading to cell apoptosis [47]. The TEM visualization in this study showed that the SSC mitochondria swelled and vacuolated during cryopreservation; however, the addition of melatonin alleviated significantly this situation. The cytochrome C content in the mitochondria or the cytoplasm was determined separately. The results showed that the expression of cytochrome C in the cytoplasm of the melatonin group cells was significantly lower than that in the control group cells; on the other hand, the expression of cytochrome C in the melatonin group mitochondria was significantly higher than that in the control group mitochondria. This indicates that melatonin reduced effectively the release of cytochrome C from the mitochondria to the cytoplasm. Our results showed that melatonin reduced SSC apoptosis caused by intrinsic apoptotic pathways. The mechanism through which melatonin regulates the mitochondrial membrane permeability is by reducing the production of ROS and regulating the expression of Bax and Bcl-2. This reduces the cytochrome C entry into the cytoplasm and effectively prevents the activation of the caspase apoptosis pathway, thereby significantly reducing SSC apoptosis during cryopreservation.

Autophagy and apoptosis cooperate to decide the fate of cells under physiological or pathological conditions [48]. Normally, autophagy removes the damaged organelles to protect the cells. However, when autophagy is excessive and dysregulated, enzymes leaking from the lysosome will initiate mitochondrial permeability, thereby promoting apoptosis [49]. In injured rat cardiac microvascular endothelial cells, melatonin downregulates the expression of autophagy-related genes *Beclin1* and *LC3-II*, and reduces the intracellular autophagosome formation. The mechanism by which melatonin protects cells entails inhibiting autophagy via the AMPK/mTOR system [50]. This corroborates our results. The TEM results showed that melatonin reduced significantly the number of autophagosomes produced during SSC cryopreservation. Moreover, the western blot results showed that melatonin reduced significantly the expression of autophagy-forming proteins (LC3-I, LC3-II, Beclin1, and ATG7) and increased significantly the expression of the receptor protein (P62). The TEM and western blot results combined suggest that melatonin reverses the freeze-induced autophagy impairment and may restore autophagy to normal levels.

## 5. Conclusions

In conclusion, the addition of 10^−6^ M of melatonin to the freezing medium increased significantly the viability of frozen-thawed SSCs, the T-AOC, and the mitochondrial membrane potential, while it reduced significantly the ROS level and the MDA content. Further research showed that melatonin reduced the oxidative damage of cells by increasing the enzyme activity and protein expression of SOD, CAT, and GSH-Px; additionally, it reduced the apoptosis caused by the intrinsic apoptotic pathway of cytochrome C and caspase, and reversed the freeze-induced excessive autophagy impairment, thereby protecting goat SSCs during cryopreservation.

## Figures and Tables

**Table 1:** Effects of different melatonin concentrations on spermatogonial stem cell (SSC) viability during cryopreservation.

Melatonin concentration (*μ*M)	0	0.01	0.1	1	10
Frozen-thawed SSC viability (%)	53.13 ± 3.68^c^	58.68 ± 2.79^bc^	62.21 ± 4.68^ab^	68.99 ± 2.78^a^	60.77 ± 3.03^bc^

Different superscript letters (a–c) indicate significant differences (*P* < 0.05). Values are expressed as the means ± SD (*n* = 3).

## Data Availability

The data used to support the findings of this study are available from the corresponding author upon request.
